# In Silico Phosphoproteomic Profiling of the MAP3K3 Signaling Network

**DOI:** 10.3390/proteomes14040050

**Published:** 2026-09-28

**Authors:** Jetna John, Suhail Subair, Leona Dcunha, Althaf Mahin, Megha Madhusudhanan, Levin John, Athira Perunelly Gopalakrishnan, Mukhtar Ahmed, Rajesh Raju

**Affiliations:** 1 Centre for Integrative Omics Data Science (CIODS), Yenepoya (Deemed to be University), Mangalore 575018, Karnataka, India; jetnajohn.study@gmail.com (J.J.); suhailsubair.ciods@yenepoya.edu.in (S.S.); leonadcunha@gmail.com (L.D.); althafmahin99@gmail.com (A.M.); meghakottapuram@gmail.com (M.M.); athirajrf@yenepoya.edu.in (A.P.G.); 2 Institute for Regeneration and Repair, University of Edinburgh, Edinburgh EH16 4II, UK; s2883886@ed.ac.uk; 3 Department of Zoology, College of Science, King Saud University, P.O. Box 2455, Riyadh 11451, Saudi Arabia; mahmed1@ksu.edu.sa

**Keywords:** MAP3K3, PsOPs, upstream kinases, substrates, carcinogenesis

## Abstract

Background: Mitogen-activated protein kinase kinase kinase 3 (MAP3K3) is a serine/threonine kinase that exhibits oncogenic activity in various cancers. Despite the established role of MAP3K3 in signal transduction, its phosphosignaling landscape, including the mechanisms underlying its activation and the identification of its upstream kinases and downstream substrates, remains poorly characterized. Methods: To explore this network, we analyzed the global phosphoproteomics datasets to identify predominant sites of MAP3K3. Co-concordance and co-occurrence analyses were conducted to characterize the functional significance of site-specific phosphorylation of MAP3K3. Results: From the 588 profiling datasets and 124 differential datasets, we identified S340, S337, and S166 as predominant phosphosites. We then categorized the phosphosites in other proteins (PsOPs), which were positively and negatively co-concordant with the predominant sites of MAP3K3. From the known and predicted upstream kinases, substrates, and interactors, we isolated the PsOPs that have a role in carcinogenesis. Conclusions: Finally, by integrating these co-concordance data with the currently known and predicted upstream kinases, substrates, and interactors, we constructed a phosphosignaling network of MAP3K3-driven carcinogenesis. This network provides insights into the potential upstream kinases and substrates of MAP3K3 in the context of carcinogenesis.

## 1. Introduction

Mitogen-activated protein kinase kinase kinase 3 (MAP3K3), also known as mitogen-activated protein kinase (MAPK)/extracellular signal-regulated kinase kinase kinase 3 (MAP3K3/MEKK3), MAPKKK3, and CCM5, was discovered by Ziegelbauer et al., who cloned the human MAP3K3 gene in 1997 from an activated peripheral blood T-lymphocyte cDNA library [1]. As a serine/threonine kinase in the MAPK superfamily [2], MAP3K3 belongs to the subfamily MAP3K, which has 24 characterized members. This subfamily includes MAP3K1 to MAP3K21, as well as A-Raf, B-Raf, and C-Raf [3]. The MAP3K3 gene, situated at chromosomal locus 17q23.3, exhibits high evolutionary conservation, with 96.5% sequence identity to mouse Mekk3. At the protein level, it translates into a 626-amino-acid polypeptide with an estimated molecular mass of 70.9 kDa. Its catalytic domain (Pkinase domain) is closely related to several other kinases, and it is conserved among all members of the MAP3K subfamily [4]. MAP3K3 also possesses a PB1 (Phox and Bem1p) domain, which is a protein–protein interaction domain. MEK5 (MAP2K5), MEKK2 (MAP3K2), and MEKK3 (MAP3K3) are the only proteins in the MAP2K and MAP3K subfamilies containing this domain. It mediates the formation of heterodimers of MEKK2 or MEKK3 with MEK5, a key regulator in the ERK5 signaling pathway [5]. MAP3K3 is predominantly localized to the cytoplasm, although immunofluorescence staining in the human U-251MG cell line has also revealed its presence in the nucleoplasm [6]. This subcellular localization is primarily initiated and regulated by various extracellular stimuli, post-translational modifications (PTMs), and interactions with specific binding partners or scaffold proteins. MAP3K3 is ubiquitously detected across human tissues, with a high abundance in the spleen, lungs, and 25 other tissues [6]. The structural framework of MAP3K3 enables it to bind both upstream activators and downstream substrates in multi-molecular complexes. Specifically, MAP3K3 forms a complex with MAP2K3, RAC1, and CCM2, and it mediates signaling by interacting with MAP2K5 and SPAG9 [7].

The biological roles of MAP3K3 include signal transduction, immune and inflammatory responses, and cellular development and growth [8]. MAP3K3 also functions as a regulator of the MAPK pathway by modulating cellular functions, including cell proliferation, differentiation, migration, and apoptosis [9]. MAP3K3 is characterized by its ability to bind ATP and metal ions, which are essential for its catalytic role as an upstream MAP kinase kinase kinase. MAP3Ks are activated either by upstream MAP4Ks or by external stimuli, such as oxidative stress, inflammatory cytokines, medications, and pressure [10]. In unstimulated cells, MAP3K3 is phosphorylated at S294, allowing it to bind to the 14-3-3 protein, thereby inhibiting its activity. When stimulated by lipopolysaccharide (LPS), a rapid reduction in phosphorylation occurs, which causes the release of MAP3K3 from protein 14-3-3, thereby restoring its active state [11]. Some studies have shown that LPS can trigger phosphorylation at S526, leading to the activation of MAP3K3 [10]. MAP3Ks activate downstream MAP2Ks, which in turn activate MAPKs [12]. The known downstream substrates of MAP3K3 include MAP2Ks (MKK3, MKK6, MKK4, and MKK5) [1,2,13,14], MAPKs (ERK and p38) [1], IRF7 (interferon regulatory factor 7) [10,15], YAP (yes-associated protein) [2], GLT1, and RCAN1 [16], which modulate cellular processes, such as apoptosis, senescence, antiviral immunity, and cancer resistance [14].

Some studies also show that the MAP3K3 can activate multiple MAPKs in vitro, such as ERK1/2 (also known as p44/42^MAPK^), JNKs (also known as c-Jun-N-terminal), p38 and ERK5, under specific conditions [1]. It activates the IκB kinase (IKK) complex, leading to NF-κB pathway activation, which helps to promote the proliferation, invasion, migration, and resistance to chemotherapy [1]. It also activates both the ERK and the stress-activated protein kinase (SAPK) pathway, which is positioned upstream of SEK (stress-activated protein kinase kinase) and MEK kinases [17]. MAP3K3 plays an important role in early embryonic cardiovascular development, endothelial cell survival, and proliferation [8]. The MAP3K3 gene is associated with several diseases, including cerebral and spinal cord cavernous malformations (CCMs and SCCMs) [18,19] and certain cancers, such as ovarian carcinoma [20], breast cancer, kidney cancer and lung cancer [8]. Somatic mutations in the MAP3K3 gene, particularly at I441M, have been linked with CCMs, which cause vascular abnormalities in the central nervous system, which can lead to the formation of neurological issues like seizures and hemorrhages [18,19]. The increased abundance of MAP3K3 is associated with cancer progression and poor survival in ovarian cancer [20]. The aberrant abundance of MAP3K3 plays an important role in the development, invasion, and metastasis of other cancers, including breast cancer, kidney cancer, and esophageal cancer. The inhibitors known are Pazopanib, which has been found to inhibit MAP3K2 and MAP3K3, providing a potential therapeutic agent for acute lung injury [21].

Although MAP3K3 is known as an oncogene in many cancers, the specific phosphorylation sites, potential upstream kinases and substrates mediating its oncogenic activity remain understudied. In this study, we focused on the comprehensive analysis of global phosphoproteomic datasets to understand the functional roles of the predominant phosphosites in MAP3K3. Additionally, we integrated predicted substrates and currently known interactors of MAP3K3 from various databases. Subsequently, a comprehensive network was constructed based on the co-phosphoregulatory patterns of the predominant MAP3K3 phosphosites with co-concordant phosphosites in other proteins (PsOPs) in predicted upstream kinases, substrates and known interactors.

## 2. Materials and Methods

### 2.1. Screening and Curation of Global Phosphoproteomics Datasets for Cataloging MAP3K3 Phosphosites

A PubMed query search was conducted to assemble the phosphoproteomics datasets, using the terms “phosphoproteomics’’ OR “phosphoproteome” NOT “Plant” NOT “Review”. We screened the published literature to compile human cellular global phosphoproteomics datasets with Class-1 MAP3K3 phosphosites, using localization probability > 75% and A-score > 13. These datasets were further categorized based on their phosphosite enrichment methods (STY/ST/Y phosphosites). Each dataset was classified into quantitative differential datasets, which show test biological/experimental conditions versus their corresponding control, and qualitative profile datasets, which show test experimental conditions and their control as independent phosphosite profiles. The differential datasets from each study were further filtered using a *p*-value cutoff of <0.05 and a fold change of >1.3 for increased abundance and <0.76 for decreased abundance. A standard format was used to define the biological and experimental conditions of each profiling and differential dataset. Each phosphosite in the datasets was mapped to its corresponding UniProt accession (downloaded on 13 May 2023) using our in-built mapping tool to ensure uniform mapping.

### 2.2. Categorization of Predominantly Detected MAP3K3 Phosphosites from Global Phosphoproteomics Datasets

Further analysis of the class-1 MAP3K3 phosphosites extracted from global phosphoproteomics datasets was performed. The number of qualitative profile and quantitative differential datasets in which each MAP3K3 phosphosite was identified was calculated and ranked. The phosphosites with the highest frequency across all datasets were considered as predominant phosphosites. The phosphosites that showed the highest frequencies were identified in more than 18% of the total qualitative and quantitative datasets.

### 2.3. Filtering the Phosphosites in Other Proteins That Exhibited Co-Concordance with Predominant MAP3K3 Phosphosites

To identify the PsOPs that showed positive or negative co-concordance with the predominant phosphosites of MAP3K3, the quantitative differential datasets representing each of them were categorized. Due to the large number of datasets comprising different experimental conditions, biological systems, and different analysis platforms employed, the re-analysis of these raw datasets was not feasible. The differential datasets were classified as increased abundance (I) and decreased abundance (D). Those that showed increased and decreased abundance, when a MAP3K3 phosphosite was observed to have increased abundance, were considered as II and ID, respectively. Subsequently, those that showed increased and decreased abundance, when a MAP3K3 phosphosite was observed to have decreased abundance, were considered as DI and DD. Accordingly, phosphosites were paired into IIDD (phosphosite pairs that were either increased abundance or decreased abundance along with MAP3K3 phosphosites) and IDDI (phosphosite pairs that showed increased abundance when MAP3K3 phosphosites showed decreased abundance or vice versa). The IIDD category of protein phosphosites denotes positive co-concordance, while IDDI denotes negative co-concordance.

Further analysis was performed using a one-sided Fisher’s exact test (FET) based on a contingency table [22,23,24]. The datasets were filtered based on criteria, such as *p*-value < 0.05, a relative differential frequency ratio threshold of ≥10% of the observed differential frequency for each target phosphosite, (n_IIDD/n_IDDI) for positively co-concordant phosphosites, (n_IDDI/n_IIDD) for negatively co-concordant phosphosites, a minimum of three independent studies (PMID_confidence) that support positive or negative co-concordance, and a minimum of three different experimental codes (Code_confidence) that support positive or negative co-concordance. The high-confidence phosphosite pairs, which were filtered using the above criteria, were used for subsequent analysis.

### 2.4. Deciphering the Co-Occurrence Patterns of MAP3K3 Phosphorylation Sites

Phosphorylation sites that co-occur within a protein at a frequency significantly greater than random chance are considered to be functionally associated, often indicating that they are structurally or sequentially adjacent, phosphorylated by the same kinases, or involved in shared biological processes [25]. To identify functionally interdependent phosphorylation sites within MAP3K3, we analyzed the pattern of co-concordance among its phosphosite pairs. We extracted differential datasets that showed multiple MAP3K3 phosphosites in the same experimental condition. The frequencies of IIDD and IDDI were calculated separately for each pair. They were further assessed by calculating the ratio of positive co-concordance (nIIDD/nIDDI) and negative co-concordance (nIDDI/nIIDD).

### 2.5. Protein and Phosphosite-Specific Interactors of MAP3K3

The binary and complex protein–protein interactors of MAP3K3 were obtained from several databases, such as HPRD (Human Protein Reference Database) [26], BIND (Biomolecular Interaction Network Database) [27], BioGRID (Biological General Repository for Interaction Datasets) [28], IntAct (IntAct Molecular Interaction Database) [29], ConsensusPathDb release 35 (downloaded on 22 May 2023) [30], CORUM (Comprehensive Resource of Mammalian protein complexes) (downloaded on 3 March 2023) [31], and RegPhos 2.0 (Regulatory Network in Protein Phosphorylation) (downloaded on 24 May 2023) [32].

### 2.6. Mapping Phosphosites in Known and Predicted Upstream Kinases and Substrates of MAP3K3

The upstream kinases and substrates predicted by in silico tools such as NetworKIN (A Resource for Exploring Cellular Phosphorylation Networks) (downloaded on 4 January 2023) [33], AKID (Automatic Kinase-specific Interactions Detection) (downloaded on 24 May 2023) [34] and experimentally validated kinases from iKiP-DB (in vitro Kinase-to-Phosphosite database) [35] were extracted. Additionally, upstream kinases and substrates predicted by Johnson et al. using a synthetic peptide library-based screening approach with a cutoff of ≥90% were also incorporated in this study [36].

### 2.7. Gene Enrichment Analysis

The functional enrichment analysis was performed using the g:GOSt module of gProfiler (https://biit.cs.ut.ee/gprofiler/gost) (accessed on 27 November 2025) [37]. The analysis was conducted using the default parameters, consisting of the cumulative hypergeometric test for enrichment, g:SCS multiple testing correction, default background including all annotated protein-coding genes of the selected organism, and an unordered gene list.

### 2.8. Conservation Analysis

Clustal Omega (https://www.ebi.ac.uk/jdispatcher/msa/clustalo) (accessed on 13 July 2026) [38] was used to perform multiple sequence alignment of MAP3K3 predominant phosphosites across multiple species. The FASTA sequences for each species were obtained from UniProt (https://www.uniprot.org/) (acessed on 13 July 2026) [7].

### 2.9. Data Visualization

M2Viz tool (https://www.ciods.in/m2viz) (accessed on 31 October 2025) [39] was used for lollipop plot data representation. Cytoscape 3.10.4 [40] was used for the interaction map visualization, and RAWGraph 2.0 (https://doi.org/10.1145/3125571.3125585) (accessed on 15 December 2025) was used for dendrograms and bar graphs. The overall workflow and findings of this study are illustrated in Figure 1.

## 3. Results

### 3.1. Phosphorylation Dynamics of MAP3K3 from Global Cellular Phosphoproteomics Datasets

To analyze the functionally significant phosphosites associated with MAP3K3, we screened published articles on human phosphoproteomics and gathered 588 profiling datasets and 124 differential datasets that contained class-1 phosphosites of MAP3K3 (Supplementary Table S1A,B). Phosphorylation of MAP3K3 was detected at 49 phosphosites in profiling datasets, whereas 19 phosphosites were identified in differential datasets.

These phosphosites detected across different experimental/biological conditions were ranked based on the frequency of each phosphosite across differential datasets. The most frequently detected phosphosites were S340, S337, and S166, with frequencies of 59, 53, and 30, respectively. These phosphosites were considered the predominant phosphosites of MAP3K3. The class-1 MAP3K3 phosphosites along with the predominant sites identified in the global human cellular profile and differential datasets are represented in Figure 2a,b. Based on the positions of these predominant phosphosites relative to the catalytic domain, MAP3K3 is classified as ‘predominant phosphosites outside the kinase domain’ (PoKD). This classification is defined within the PxKD database [Predominant-Phosphosite (classified) in kinases based on their distribution in primary sequence] [41]. The site-specific phosphorylation events reported at S166 and S337 are mediated by the kinase SGK1 (serum- and glucocorticoid-induced kinase 1), which is induced by various extracellular stimuli, such as tumor necrosis factor-alpha (TNF-α), arsenite, forskolin, and serum [42,43].

The evolutionary conservation analysis across species revealed that human MAP3K3 is highly conserved across all species except zebrafish. The predominant phosphosites of human MAP3K3 are also conserved among species except zebrafish. The predominant phosphosites S340 and S337 of human MAP3K3 are conserved in the mouse, rat, and bovine orthologs of MAP3K3 but are not conserved in zebrafish. The predominant phosphosite S166 of human MAP3K3 is conserved in mouse, rat, and bovine orthologs, and S169 of zebrafish orthologs. The conservation analysis is represented in Figure 2c.

### 3.2. Quantitative Co-Concordance Analysis of Predominant MAP3K3 Phosphosites

The co-concordance analysis was performed to categorize the PsOPs that are co-concordant with the predominantly detected phosphosites. The datasets were first classified according to increased abundance (I) and decreased abundance (D) patterns parallel to the phosphorylation status of these predominant sites. They were further labeled as IIDD (positive co-concordance) and IDDI (negative co-concordance). To accomplish this, strict inclusion and exclusion criteria were applied, which were based on a contingency table, followed by an FET analysis. The criteria include a *p*-value of <0.05 (Supplementary Table S2A–F), a ≥10% frequency cutoff for the PsOPs in positive or negative co-concordance with MAP3K3 predominant sites, a ≥3 PMID_confidence and a ≥3 Code_confidence. After applying these cutoffs, MAP3K3 predominant site S340 showed positive co-concordance with 604 PsOPs and negative co-concordance with 222 PsOPs (Supplementary Table S2G,H). Similarly, the phosphosite S337 showed positive co-concordance with 1033 PsOPs and negative co-concordance with 77 PsOPs (Supplementary Table S2I,J), while the phosphosite S166 showed positive co-concordance with 1457 PsOPs and negative co-concordance with 92 PsOPs (Supplementary Table S2K,L).

Among the co-concordant PsOPs, MAP3K3_S340 and RANBP10_S369 showed top positive co-concordance with a frequency of 18 and BAD_S99 showed top negative co-concordance with a frequency of 16. Studies have shown RANBP10 to be involved in microtubule modulation and cancer progression [44,45], whereas BAD, a pro-apoptotic member of the Bcl-2 family, promotes cell death [46]. Similarly, among PsOPs of MAP3K3_S337, NELFA_T277 and AHCTF1_S1232 showed top positive co-concordance with a frequency of 25 and TJP1_S353 and SRSF10_S131 showed top negative co-concordance with a frequency of 11. NELFA is a subunit of the NELF complex that regulates gene transcription [47], while AHCTF1 is a multi-domain nucleoporin essential for the reassembly of the nuclear pore complex (NPC) on chromatin following mitosis (cell division) [48]. TJP1 maintains cellular barriers and polarity by linking transmembrane proteins and the actin cytoskeleton [49], while SRSF10 regulates pre-messenger RNA (pre-mRNA) splicing [50]. Meanwhile, PRRC2B_S416 showed top positive co-concordance with a frequency of 21, and RANBP2_T1396 showed top negative co-concordance with a frequency of 11 with MAP3K3_S166. PRRC2B is a novel m6A reader protein that binds to specific mRNAs by influencing processes like alternative splicing and mRNA decay [51,52], while RANBP2 is the largest nucleoporin of the NPC, functioning as a gatekeeper that regulates transport between the cytoplasm and the nucleus [53]. The top 10 PsOPs that are positively and negatively co-concordant with the predominant sites of MAP3K3 are represented in Figure 3.

Gene set enrichment analysis of co-concordant PsOPs highlighted their role in many biological processes, including chromatin organization, DNA repair, cell division, cell cycle dynamics, cell migration, modulation of cell growth, cell differentiation, and developmental growth, which are associated with cancer. The gene ontology analysis of proteins co-concordant with the predominant sites of MAP3K3 is given in Supplementary Table S3A–F.

### 3.3. Co-Occurrence Patterns of Phosphosites Within MAP3K3

Li et al., 2017 [25] introduced a qualitative co-occurrence analysis method, demonstrating that pairs of phosphorylation sites co-occurring across a broad range of high-throughput mass spectrometry datasets are much more likely to be functionally connected than randomly selected pairs. To explore this in the context of MAP3K3, we analyzed the co-occurrence patterns of the phosphosites within the differential phosphoproteomics datasets of MAP3K3 (Figure 4). From the data, we identified that the phosphosite pairs S175 and S340, S176 and S250, and S166 and S176 tend to be positively co-occurring. The phosphosites S175 and S340 showed increased abundance together in 10 datasets and decreased abundance together in 1 dataset with a ratio of 10. The phosphosites S176 and S250 showed increased abundance together in 10 datasets and did not show any decreased abundance. The phosphosites S166 and S176 showed increased abundance together in nine datasets and decreased abundance together in one dataset. The phosphosite pair S340 and S355 were found to co-occur negatively. Although they are in proximity, they tend to be oppositely co-occurring. The co-occurrence frequency ratios of phosphosites of MAP3K3 are provided in Supplementary Table S4A. The absence of significant co-occurrence among the predominant sites suggests that phosphorylation on these sites may modulate distinct and independent functions of MAP3K3. Further research is needed to interpret the details concerning their functional relevance in MAP3K3.

### 3.4. Systematic Cataloging of Potential Upstream Regulatory Kinases Functionally Associated with MAP3K3

One of the most important types of post-translational modification (PTM) on proteins is phosphorylation, which is mediated by protein kinases. Only a few upstream kinases have been known to phosphorylate MAP3K3. Thus, to identify additional potential upstream kinases, we integrated the kinase–substrate data from Johnson et al. [36] and predicted data using predictive tools such as NetworKIN [33] and AKID [34] into the co-concordant protein phosphosite list of MAP3K3. Using this list of potential upstream kinases, we screened the co-concordance of their phosphosites with respect to predominant phosphosites of MAP3K3. Consequently, we identified two phosphosites that were positively co-concordant and two that were negatively co-concordant with S340 of MAP3K3. Similarly, 18 phosphosites in upstream kinases were found to be positively co-concordant with phosphosite S337 of MAP3K3, while no phosphosites were found to be negatively co-concordant. MAP3K3_S166 showed 54 and 3 phosphosites within predicted upstream kinases that exhibit positive and negative co-concordance, respectively. The high-confidence phosphosites in upstream kinases of MAP3K3 are given in Figure 5 (Supplementary Table S4B).

Among the 18 phosphosites in upstream kinases that are co-concordant with MAP3K3_S337, 12 were predicted by Johnson et al. [36]. These include phosphosites of PKN1_S916, RPS6KA5_S376, and RPS6KA3_S386 that show involvement in the activation mechanism and enzymatic activity. Among the co-concordant phosphosites of MAP3K3_S337, five were predicted as upstream kinases by in silico tools, and their sites that are co-concordant with MAP3K3 include MAP2K2_T394, PRKCE_S368, MAPK3_Y204, HIPK2_S827, and MAPK3_T202. From these, MAP2K2_ T394, MAPK3_Y204 and T202 are associated with the induction of enzymatic activity and show positive co-concordance at S337 of MAP3K3. From the 54 co-concordant phosphosites in upstream kinases of MAP3K3_S166, 34 of them were predicted by Johnson et al. [36], of which two of them, such as RPS6KA1_S380 and MAP2K2_T394, induce enzymatic activity. In the remaining phosphosites, 18 were predicted by in silico tools, of which 7 of them, such as MAP2K2_T394, ARAF_S257, BRAF_S446, MAPK1_T185, MAPK1_Y187, PAK2_S141, and PAK1_T212, show induction of enzymatic activity.

Potential upstream kinases have a role in carcinogenesis and are positively co-concordant with the predominant sites S337 and S166. They include PKN1_S916, PRKCE_S368, MAPK3_Y204 and T202, SRPK2_S494 and S497, RPS6KA1_S380, RAF1_S259, PAK4_S181, BRAF_S446, MAPK1_T185 and Y187, PAK2_S141, and PAK1_T212.

### 3.5. Systematic Cataloging of Potential Substrates Functionally Associated with MAP3K3

Currently known substrates of MAP3K3 include MAP2K3 (MKK3), MAP2K6 (MKK6), MAP2K4 (MKK4) [1,14], MAP2K5 (MKK5) [2,13], ERK, p38 [1], IRF7 [10,15], YAP [2], GLT1, and RCAN1 [16]. Among them, only MAP2K4 was observed to be co-concordant with the predominant phosphosites of MAP3K3. To identify additional potential substrates that could be phosphorylated by MAP3K3, we integrated the substrates predicted by in silico tools as well as those categorized by Johnson et al. with the co-concordant datasets [36]. The list of potential substrates was analyzed with the abundant co-concordance of predominant phosphosites in MAP3K3. Analysis of the co-concordance data for MAP3K3_S340 revealed that 26 predicted substrates exhibited positive co-concordance, while 15 showed negative co-concordance based on the Johnson et al. dataset [36]. Among these, FRYL_T1959, GIT1_S385, MELK_S505, and CASC3_S125 showed the highest positive co-concordance, whereas TPD52L2_S161, DSP_S1127, and DOCK6_S1308 showed the highest negative co-concordance. Similarly, MAP3K3_S337 showed positive co-concordance with 57 substrates predicted from high-throughput data and Johnson et al. datasets [36], with MAP3K3_S166 and NELFA_T277 showing the highest co-concordance frequencies. This site also showed negative co-concordance with four substrates predicted by Johnson et al. [36], with SRSF10_S131 showing the highest negative co-concordance frequency. For MAP3K3_S166, 94 phosphosites in substrates predicted by high-throughput data and Johnson et al. [36] showed positive co-concordance with MAP3K3_S337, where RBM15B_S562 and PROSER2_S43 showed the highest co-concordance frequencies. Furthermore, this site also showed negative co-concordance with eight substrate phosphosites predicted by Johnson et al. [36], among which ZBTB21_S345 showed the highest co-concordance frequency. The phosphosites in substrates that are positively and negatively co-concordant with the predominant sites of MAP3K3 are illustrated in Figure 6 (Supplementary Table S4C).

### 3.6. Phosphosignaling Network of MAP3K3-Driven Carcinogenesis

The co-concordant PsOPs in potential upstream kinases and substrates with activities associated with cancer, such as apoptosis, cell proliferation, migration, cell growth, and carcinogenesis, were screened and illustrated to form the phosphosignaling network of MAP3K3-driven carcinogenesis. Based on the evidence that silencing MAP3K3 showed inhibition of cell proliferation, migration, and invasion in lung cancer cell lines [54], along with other MAP3K3 studies in ovarian cancer, breast cancer, kidney cancer, and esophageal cancer [20], we filtered 16 phosphosites of potential upstream kinases and 13 phosphosites of potential substrates with established roles in cancer-related biological processes, as illustrated in Figure 7 (Supplementary Table S4D,E). Among the potential upstream kinases and downstream substrates, few were also identified as binary interactors of MAP3K3. They include PAK1_T212, MAPK3_Y204, and T202, MAPK1_T185, Y187 and MAP2K4_S80. Thus, we propose that these PsOPs could have major roles in modulating the carcinogenic roles of MAP3K3.

### 3.7. Co-Concordance of Predominant MAP3K3 Phosphosites with Known Binary and Complex Interactors

To analyze the interactors co-concordant with the predominant sites of MAP3K3, we assembled a list of its binary and complex interactors from publicly available databases, such as HPRD [26], BIND [27], BioGRID [28], ConsensusPathDb release 35 [30], CORUM [31], and RegPhos 2.0 [32]. Subsequently, we identified 14 and 7 phosphosites within known binary interactors that exhibited positive and negative co-concordance, respectively, with the predominant MAP3K3 phosphosite S340. Similarly, the S337 site was associated with 39 positively and 4 negatively co-concordant phosphosites among its known binary interactors. In contrast, the S166 site was associated with 62 phosphosites within known binary interactors, showing exclusively positive co-concordance. The co-concordant PsOPs of MAP3K3 that show binary interaction are illustrated in Figure 8 (Supplementary Table S4F). Among the known binary interactors of MAP3K3, 25 were predicted as upstream kinases and 14 were predicted as substrates by various in silico tools.

We found that WNK1_S2372 was positively co-concordant with all three predominant sites of MAP3K3. Additionally, WNK1 phosphosites S2370 and S2032 also showed positive co-concordance with MAP3K3_S340 and MAP3K3_S166, respectively. NEDD4L shows positive co-concordance in S487 and T367 with MAP3K3_S340 and MAP3K3_S166, respectively, while NEDD4L_S449 shows negative co-concordance with MAP3K3_S340. We found that S281 of RAI14 shows positive co-concordance with MAP3K3_S340, while S296 and S381 of RAI14 show positive co-concordance with both S337 and S166 of MAP3K3. Additionally, MAP3K3_S337 shows positive co-concordance with S300 of RAI14, and MAP3K3_S166 shows positive co-concordance with T297 and S293 of RAI14. LATS1_S464 also showed positive co-concordance with MAP3K3_S166. The site S613 of LATS1 shows positive co-concordance with the other predominant sites of MAP3K3, such as S340 and S337. MPRIP shows positive co-concordance in S220, S224, and S891 with MAP3K3_S337, and it also shows positive co-concordance in S265, S619, and S891 with MAP3K3_S166. The phosphorylation sites S329, S705, and S268 of SPAG9 showed positive co-concordance with S337 of MAP3K3, and the sites T217, S594, T276, and S730 showed positive co-concordance with S166 of MAP3K3. VCP_S197 shows positive co-concordance with MAP3K3_S337. MAP2K4_S80, MATR3_S188, and IQGAP2_S16 show positive co-concordance with MAP3K3_S166.

Our study also identified a set of complex interactors that interact with the predominant sites of MAP3K3 (Supplementary Table S4G). The phosphosite-level study shows positive and negative co-concordance with many proteins. Specifically, MAP3K3_S340 shows positive and negative co-concordance with 139 and 30 PsOPs, respectively. Similarly, the site S337 of MAP3K3 shows 141 positively and 9 negatively co-concordant PsOPs. For S166 of MAP3K3, it shows positive and negative co-concordance with 257 and 18 PsOPs. Among these, we assessed the co-concordant PsOPs which have known biological roles. PsOPs show association mainly in cell growth, motility, carcinogenesis, apoptosis, control of cell cycle, and signaling pathway modulation.

## 4. Discussion

MAP3K3 (MEKK3) is an understudied kinase, particularly in the context of its upstream kinases and substrates that mediate its role in carcinogenesis. While the key signaling pathways of MAP3K3, such as the MAPK signaling pathway, JNK, NF-κB, p38, AKT, and ERK5 pathways are known, there is a lack of comprehensive understanding of its substrate repertoire [5,8,55]. Previous studies have investigated several MAP3K3 phosphorylation sites, including S526, T294, S166, and S337. The autophosphorylation site S526 of MAP3K3 within the activation loop (T-loop) is known to be regulated by the phosphatase PP2A and the scaffolding protein 14-3-3 [56]. The LPS-induced dephosphorylation of MAP3K3 at T294 facilitates NF-κB activation by releasing MAP3K3 from the inhibitory binding of the 14-3-3 protein [11]. Adams et al. demonstrated that various stimuli such as TNF, arsenite, forskolin, and serum promote phosphorylation of MAP3K3 at S166 and S337 [43]. The phosphorylation of MAP3K3 at S166 and S337 by SGK1 inhibits MEKK3-MKK3/6 signal transduction [42]. This embraces the view of phosphosite-specific associations of MAP3K3.

To identify more potential upstream kinases and substrates of MAP3K3 that may contribute to carcinogenesis, we analyzed large-scale phosphoproteomic datasets. We identified three phosphosites (S340, S337, and S166) as predominant, with high frequencies across qualitative and quantitative datasets under various experimental conditions. We further observed that these predominant phosphosites do not co-occur across experimental conditions, suggesting that they were not phosphorylated simultaneously and may independently contribute to the modulation of MAP3K3 function.

To further understand the significance of their functions, we analyzed high-confidence PsOPs that are positively or negatively co-concordant with the predominant sites of MAP3K3. We then integrated the known and predicted data on upstream kinases, substrates, and binary interactors. From these, we identified a few potential upstream kinases and substrates that have a role in carcinogenesis.

Previous research has established the oncogenic role of MAP3K3 across several malignancies, including nasopharyngeal carcinoma [57], ovarian cancer [20], lung cancer [8], and breast cancer [54]. In addition to cancer, MAP3K3 is also associated with CCMs, SCCMs [18] and verrucous venous malformations (VVMs) [58]. The knockdown study of MAP3K3 in breast cancer inhibited cell proliferation and colony formation. It is also a potential therapeutic target in breast cancer [54]. The silencing of MAP3K3 in lung cancer cell lines using siRNA decreased cell proliferation, migration, and invasion. It was also associated with a decrease in JNK, p38, AKT, and GSK3β (Glycogen Synthase Kinase 3 beta) pathways [8]. Based on this evidence, we identified a few potential upstream kinases of MAP3K3 that play a role in carcinogenesis and are also concordant with the predominant sites of MAP3K3, such as PKN1, RPS6KA1, SRPK2, PRKCE, PAK4, PAK2, RAF1, and BRAF. Among these, PKN1 is known to promote cell proliferation and invasion in lung cancer [59]. RPS6KA1 also promotes cell proliferation in prostate cancer and promotes malignant progression of glioma [60]. SRPK2 promotes cell proliferation, migration, invasion, growth and cell cycle progression in prostate cancer, pancreatic cancer, and colon cancer [61,62,63]. PRKCE promotes cell survival, epithelial-to-mesenchymal transition, and chemoresistance across prostate, breast, lung, and gallbladder malignancies [64,65,66,67,68,69]. PAK4 and PAK2 promote cell migration, invasion, and proliferation across multiple cancers [70,71]. Additionally, PAK4 has been found to be elevated in esophageal squamous cell carcinoma cells, gastric cancer cells, ovarian cancer cells, and cervical cancer tissues [71]. The autophosphorylation of PAK2 at S141 is required for optimal kinase activity and reduces its interaction with its upstream activator, the small GTPase Cdc42 [72]. RAF1 is a proto-oncogene that activates the MEK-ERK pathway and promotes cell proliferation and survival [73]. MAP3K3 helps in YAP-dependent BRAF inhibitor resistance in melanoma cells [2]. Some of the potential upstream kinases are also known to be interactors of MAP3K3, such as PAK1, MAPK1, and MAPK3. PAK1 is known to be associated with cell motility, survival, proliferation, and cell cycle regulation [74]. Additionally, PAK1 at T212 is phosphorylated by cell cycle-modulated kinases Cdc2 and Cyclin-dependent kinase 5 (Cdk5) [75]. The dual phosphorylation of MAPK1 at T185 and Y187 leads to the hyperactivation of the MAPK pathway, promoting oncogenic processes like enhanced cell growth, migration, invasion, and drug resistance [76]. MAPK3 is a known oncogenic factor in several cancers, such as breast, ovarian, colorectal, liver, lung, thyroid, and gastric cancers. In such cancers, increased abundance of MAPK3 is associated with initiation, progression, cancer cell metastasis, and drug resistance [77]. The phosphorylation of Y204, along with T202 of MAPK3, is required for the full enzymatic activity of MAPK3. The MAPK3 activation is the final step in the RAF/MEK/ERK signaling cascade, which is frequently hyperactivated in cancer [78].

The potential downstream substrates of MAP3K3 that have a role in carcinogenesis and are also positively or negatively co-concordant with the predominant sites of MAP3K3 were categorized. Among these, knockdown of SMC1A is known to inhibit cell proliferation, growth, and migration in colorectal cancer, prostate cancer, and hepatocellular carcinoma [79,80,81]. The increased abundance of KIF4A mediates tumor progression and invasion in lung cancer and glioma [82]. It was also found to promote proliferation of osteosarcoma cells through the MAPK signaling pathway [83]. PLK1 is an oncogene that showed increased abundance in many cancers, such as glioma, colorectal cancer, ovarian carcinoma, breast cancer, and prostate cancer [84]. Studies have shown increased abundance of the MFF protein in numerous cancers, especially non-small cell lung cancer (NSLC) [85,86]. INCENP shows increased abundance in breast tissues [87]. EIF3A is a proto-oncogene, and its increased abundance promotes cancer cell proliferation [88]. One of the potential downstream substrates, MAP2K4, is also known to be an interactor of MAP3K3. Phosphosite S80 of MAP2K4, an experimentally validated substrate, shows positive co-concordance with MAP3K3_S166. MAP2K4 is a tumor suppressor gene that is frequently found to be deleted in various cancer types, like ovarian serous carcinoma, ovarian endometrioid carcinoma, pancreatic carcinoma and lung carcinoma [89,90,91,92]. However, the S80 phosphorylation site is an inhibitory site that inhibits its activity and apoptosis, resulting in cancer.

Through this systemic integration of known and predicted upstream kinases and substrates, we mapped a comprehensive MAP3K3 phosphosignaling network that underscores its central role in driving carcinogenesis. Despite MAP3K3 being widely recognized as an oncogene in most malignancies, there is a significant gap in the literature regarding phosphosite-specific functional studies in the context of cancer [8]. So, our study can be used as a foundation for future experimental studies into the potential significance of novel substrates and upstream kinases in cancer. Further studies on the impact of these phosphorylation with MAP3K3 are needed to elaborate on their relevance in cancer.

Additionally, to experimentally validate this in silico study, future studies on mapping the MAP3K3 interactome and kinase–substrate networks across specific cellular compartments and diverse stress conditions using high-resolution workflows are needed [93,94]. Implementing advanced in vivo proximity labeling strategies, such as the combination of endogenous CRISPR-tagged TurboID with Data-Independent Acquisition mass spectrometry, will be essential for mapping the weak and transient kinase–substrate interactors that define this network [95,96]. Recent spatiotemporal studies in model systems illustrate how core kinases rapidly rewire interactors during baseline development and acute stress environments [97]. Furthermore, incorporating an advanced dual-channel learning framework and state space modeling that extracts macro-topological graph structures and micro-semantic subgraphs in the future gives more confidence to our co-concordance study and predicts interactions and multi-layered regulatory networks across specific disease conditions [98]. Applying these high-resolution pipelines to MAP3K3 will be critical to experimentally confirm our predicted kinase–substrate network and how it reorganizes during carcinogenesis.

## 5. Limitations of the Study

While this study maps the comprehensive phosphosignaling network of MAP3K3, our analysis aggregates modification events that may exist on distinct proteoforms rather than co-occurring on a single protein molecule. Consequently, our findings capture a macro-level view of MAP3K3 modification patterns but cannot distinguish between individual proteoform states. Resolving this molecular diversity will require future studies utilizing top-down proteomics or targeted, proteoform-specific mass spectrometry workflows. Furthermore, although we implemented strict inclusion and exclusion criteria, including a localization probability threshold of ≥75%, an A-score ≥ 13, and a statistical *p*-value, the use of public phosphoproteomic datasets introduces structural heterogeneity. Because these source data span highly diverse experimental platforms, cell lines, and biological conditions, absolute normalization is structurally constrained without uniform raw data reprocessing. Thus, these data-driven insights should be considered high-confidence candidates that require independent validation. Finally, the upstream kinases and substrate candidates of MAP3K3 reported here rely heavily on predictive algorithms. Computational tools like NetworKIN and PhosphoSitePlus frequently assign kinase–substrate relationships based on broader pathway associations or spatial co-concordance. To establish these networks more robustly, these candidate regulators and substrates must be validated through targeted phosphomotif enrichment and concordant, co-abundant phosphorylation profiles across diverse experimental models.

While this study focuses on candidate phosphorylation sites and their kinase–substrate networks associated with MAP3K3-driven carcinogenesis, it does not identify the functional consequences of individual phosphorylation events. How specific phosphosites regulate MAP3K3 kinase activity, protein stability, subcellular localization, and interaction with signaling partners remains unresolved. Consequently, the absence of direct experimental validation is a primary limitation of this work; targeted biochemical, cellular, and in vivo assays remain necessary to translate these in silico predictions into concrete biological mechanisms.

## 6. Conclusions

In conclusion, our study contributes to the understanding of the MAP3K3-driven carcinogenesis signaling network by identifying potential upstream kinases and substrates of MAP3K3. Here, we focused on the predominant sites S340, S337, and S166, which were identified across several experimental conditions. First, we analyzed the PsOPs that were positively or negatively co-concordant with these predominant sites, as well as the co-occurrence pattern of phosphosites within MAP3K3. We then explored the potential upstream kinases and substrates of MAP3K3 that also exhibit binary interactions. Finally, we integrated these findings to construct a MAP3K3-driven carcinogenesis signaling network.

## Figures and Tables

**Figure 1:**
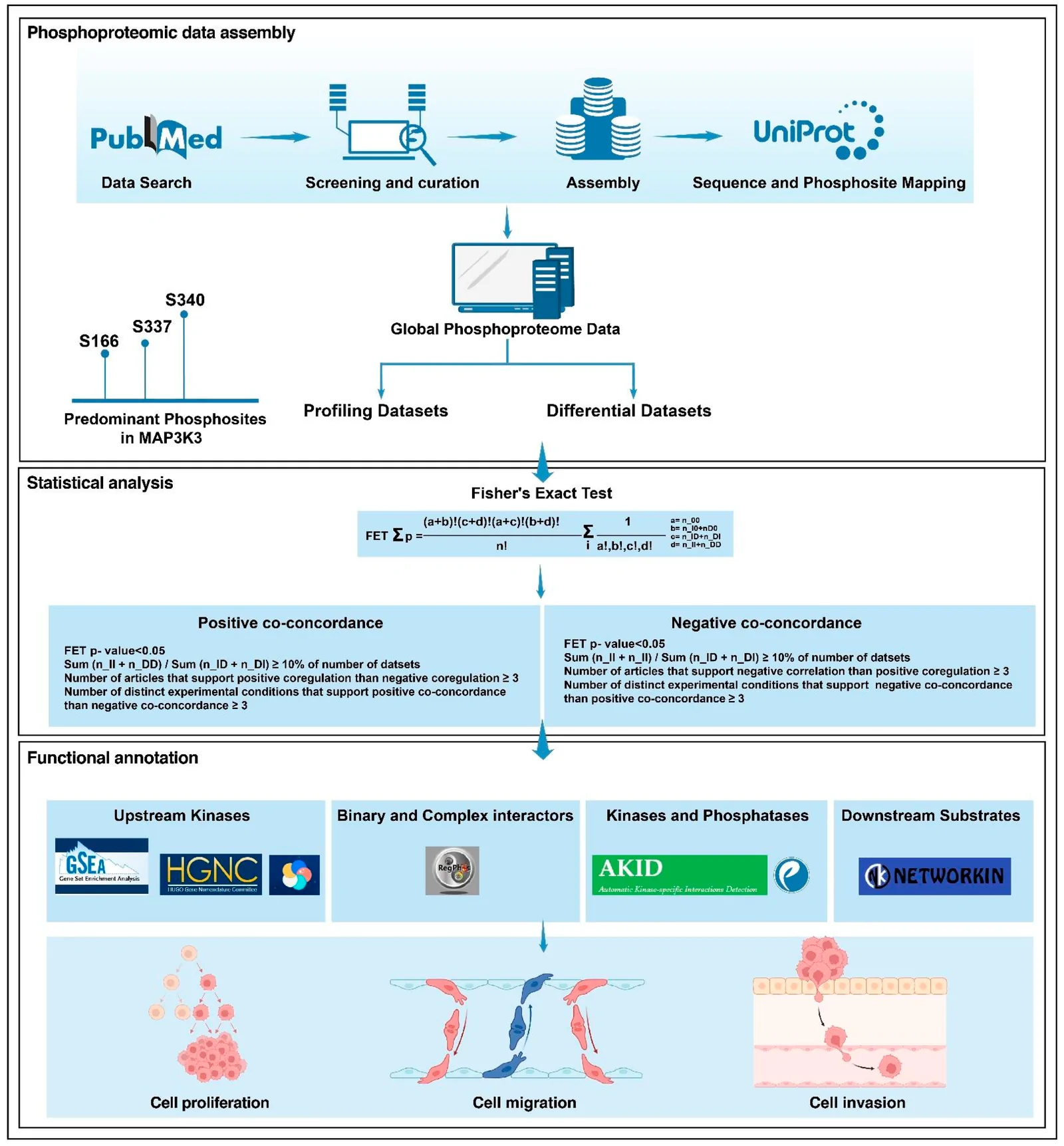
Workflow and findings of the study, including data assembly and analysis of phosphosites in MAP3K3 and their co-concordant phosphosites in other proteins.

**Figure 2:**
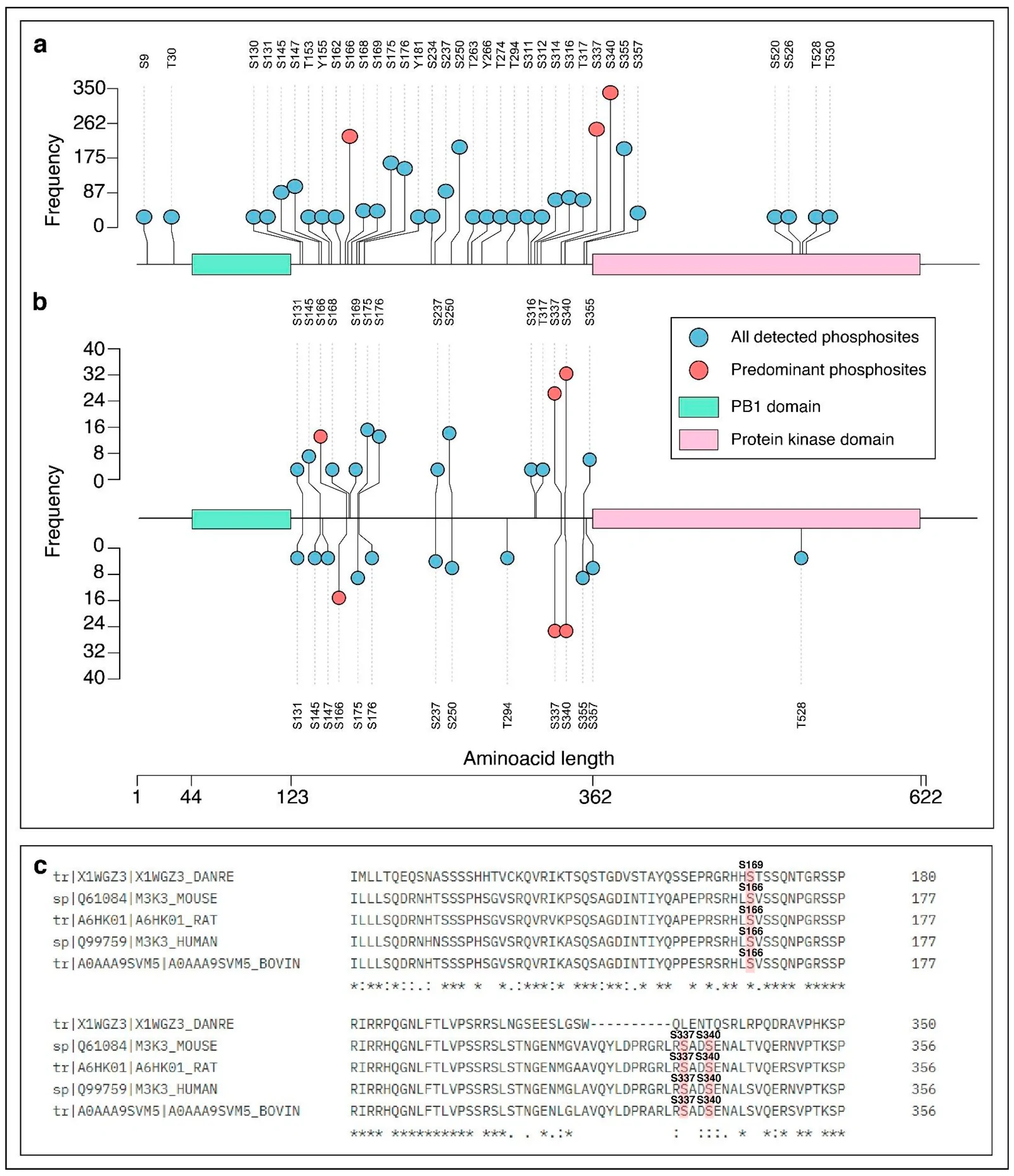
Lollipop plot illustrates the phosphosites and predominant sites of MAP3K3 identified from global human cellular profile and differential datasets using mass spectrometry. The *X*-axis represents the amino acid sequence length, and the *Y*-axis represents the frequency of phosphosites. The predominant phosphosites are highlighted in red. (**a**) Class-1 phosphosites of MAP3K3 in the profile dataset. (**b**) Class-1 phosphosites of MAP3K3 in the differential dataset. (**c**) Evolutionary conservation of human MAP3K3 predominant phosphosites across species. The predominant phosphosites and the corresponding conserved residues are highlighted in red (MAP3K3_Zebrafish, MAP3K3_Mouse, MAP3K3_Rat, MAP3K3_Human, and MAP3K3_Bovine). An * (asterisk) represents fully conserved residues, : (colon) represents conservation between strongly similar properties, and . (period) represents conservation between weakly similar properties.

**Figure 3:**
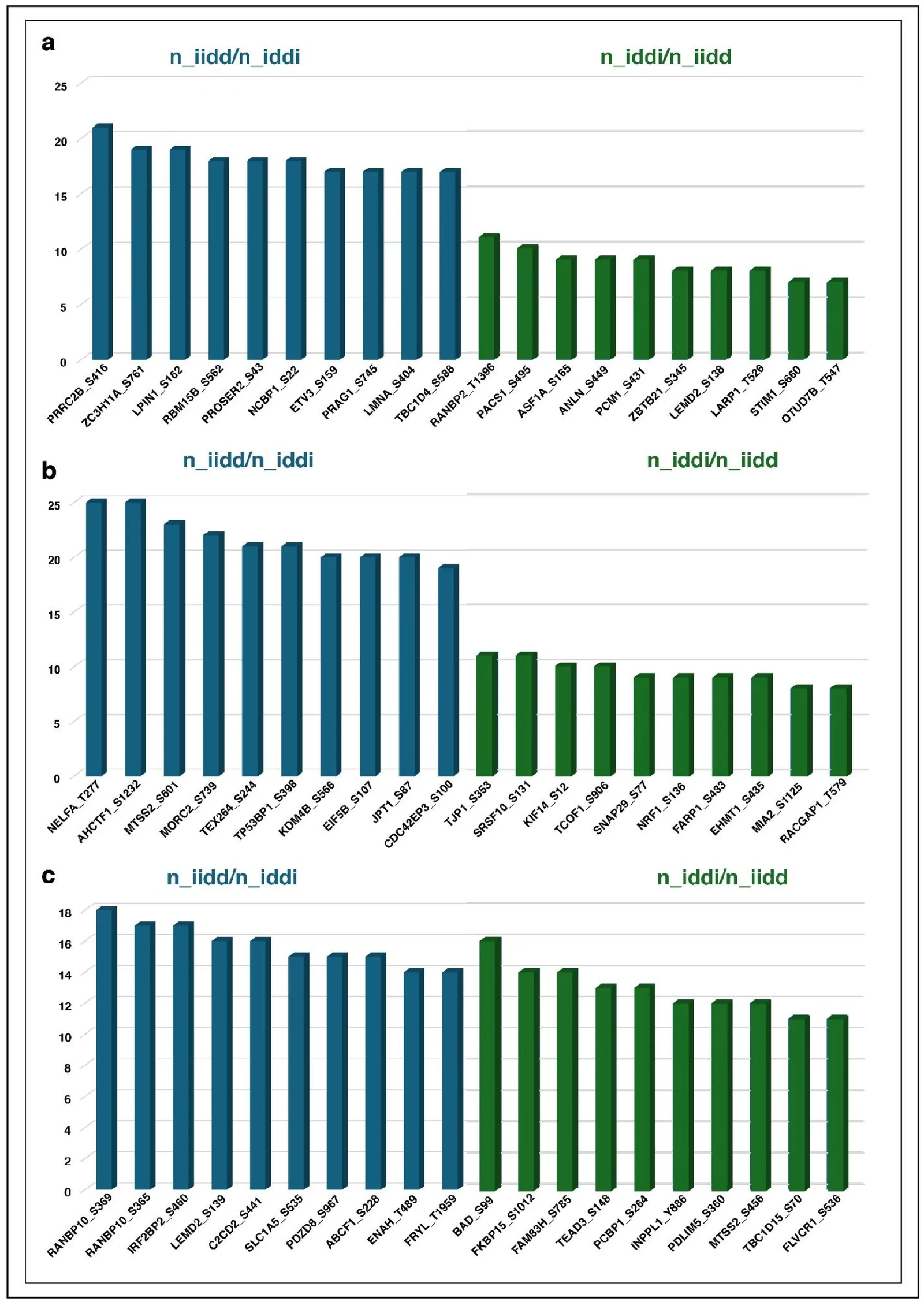
Bar diagram representing top 10 PsOPs that show positive and negative co-concordance with the predominant sites of MAP3K3. The blue bars represent the positively co-concordant PsOPs and the green bars represent the negatively co-concordant PsOPs. (**a**) Top 10 positively and negatively co-concordant PsOPs of MAP3K3 at S166. (**b**) Top 10 positively and negatively co-concordant PsOPs of MAP3K3 at S337. (**c**) Top 10 positively and negatively co-concordant PsOPs of MAP3K3 at S340.

**Figure 4:**
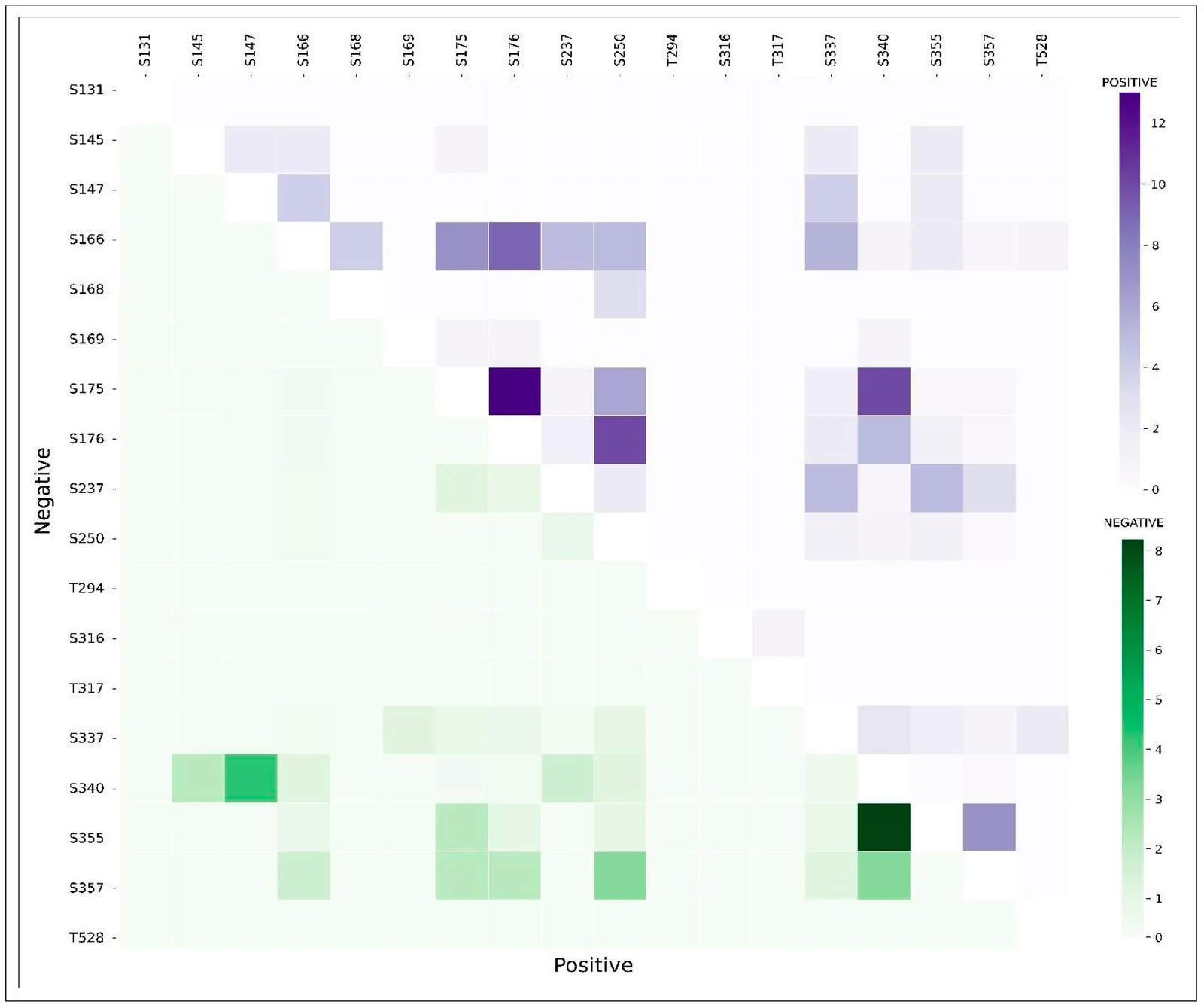
Heat map representing the co-occurrence pattern of phosphosite pairs within MAP3K3. The upper violet triangle represents the positive co-occurrence pattern, and the lower green triangle represents the negative co-occurrence pattern of phosphosite pairs within MAP3K3, with the color gradient representing the frequency of co-occurrence.

**Figure 5:**
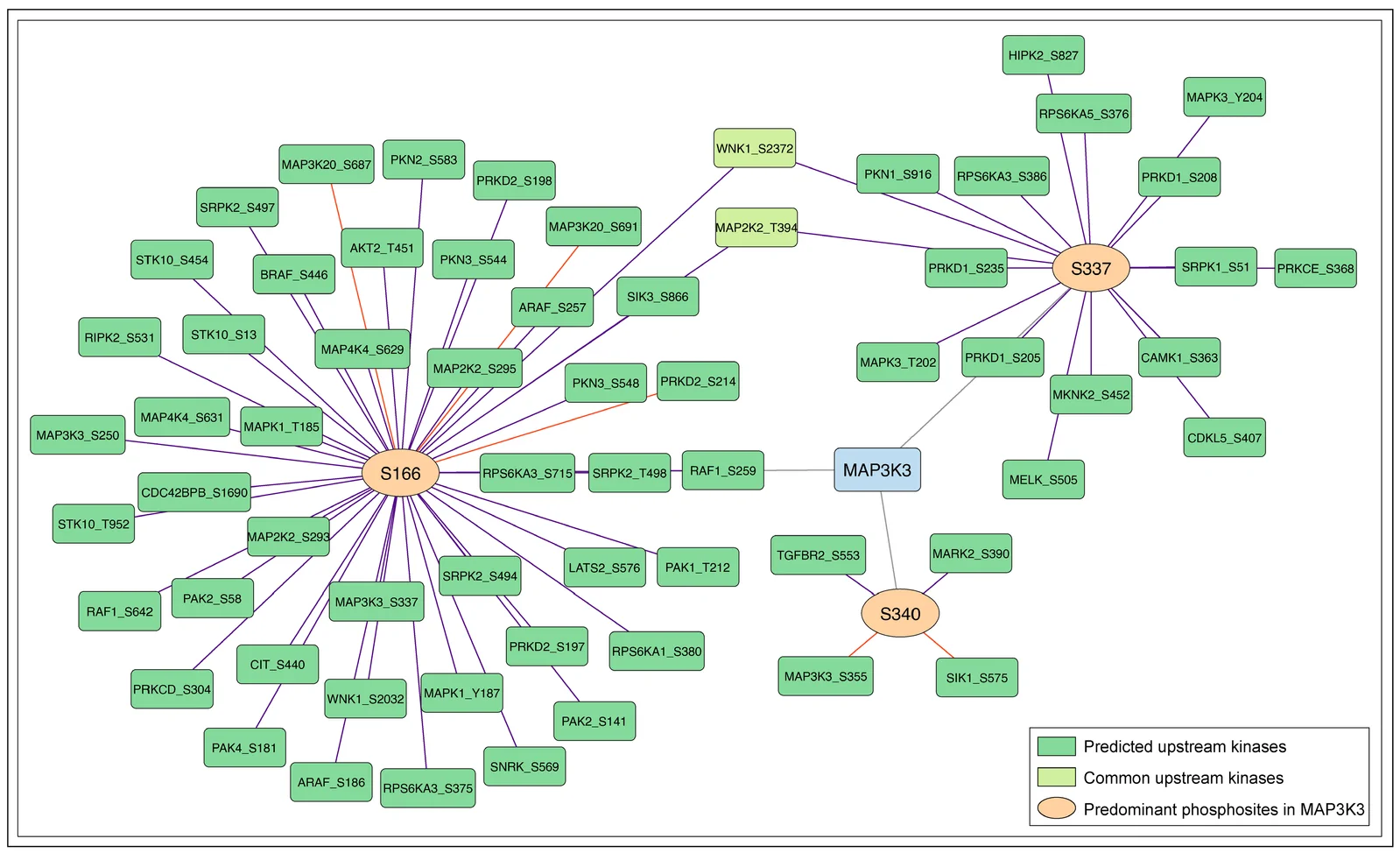
Predicted upstream kinases that are positively and negatively co-concordant among the PsOPs of the predominant sites of MAP3K3. The network is classified into upstream kinases co-concordant with MAP3K3_S166 (**left**), upstream kinases co-concordant with MAP3K3_S337 (**right**), and upstream kinases co-concordant with MAP3K3_S340 (**below**). Upstream kinases that are positively co-concordant with predominant sites of MAP3K3 (blue lines), upstream kinases that are negatively co-concordant with predominant sites of MAP3K3 (red lines). The central target protein of this study, MAP3K3, is highlighted in the blue box.

**Figure 6:**
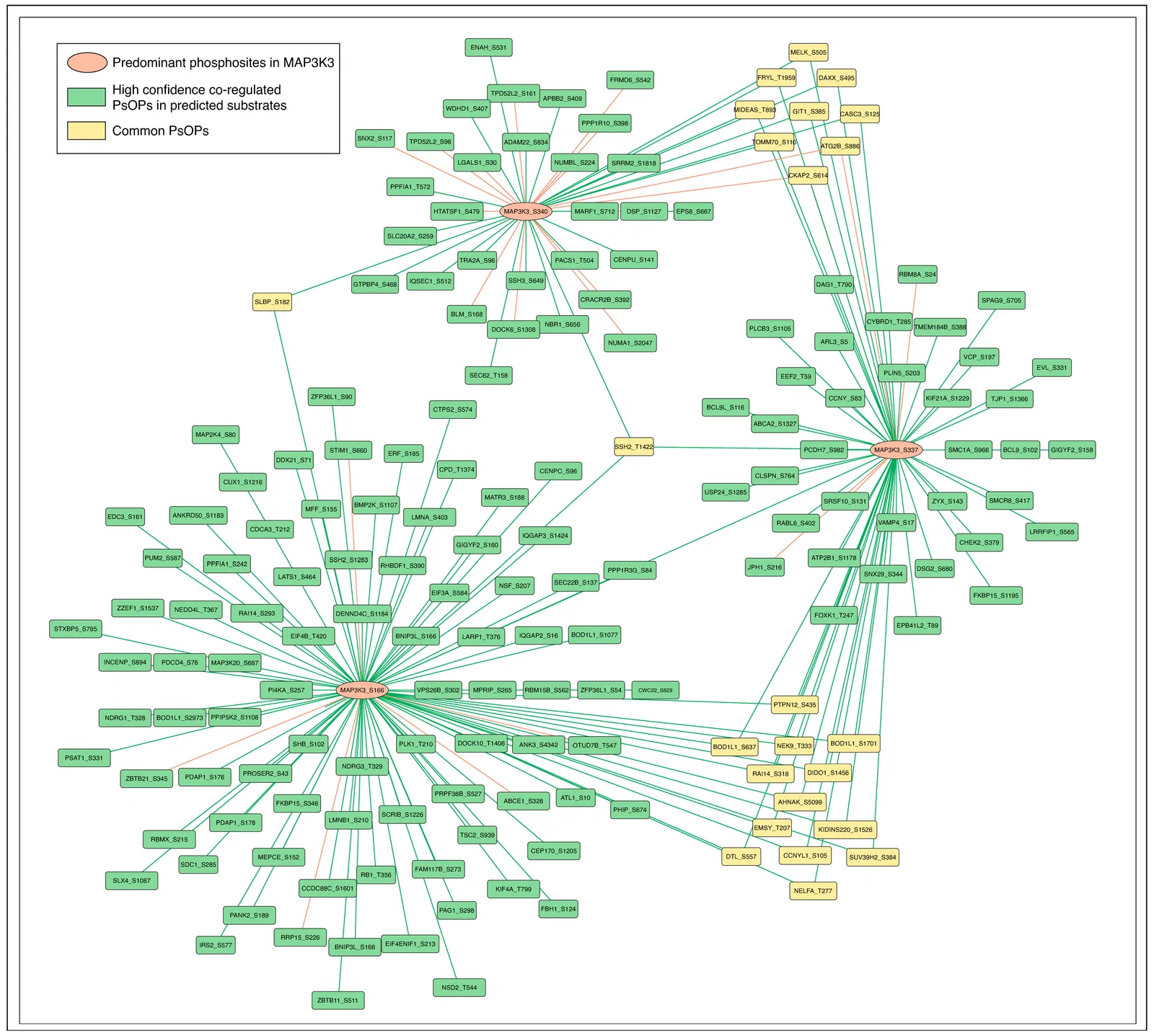
Predicted substrates that are positively and negatively co-concordant among the PsOPs of the predominant sites of MAP3K3. The network is classified into three main parts: substrates co-concordant with MAP3K3_S166 (**left**), substrates co-concordant with MAP3K3_S337 (**right**), and substrates co-concordant with MAP3K3_S340 (**above**). Substrates that are positively co-concordant with predominant sites of MAP3K3 (green lines); substrates that are negatively co-concordant with predominant sites of MAP3K3 (red lines).

**Figure 7:**
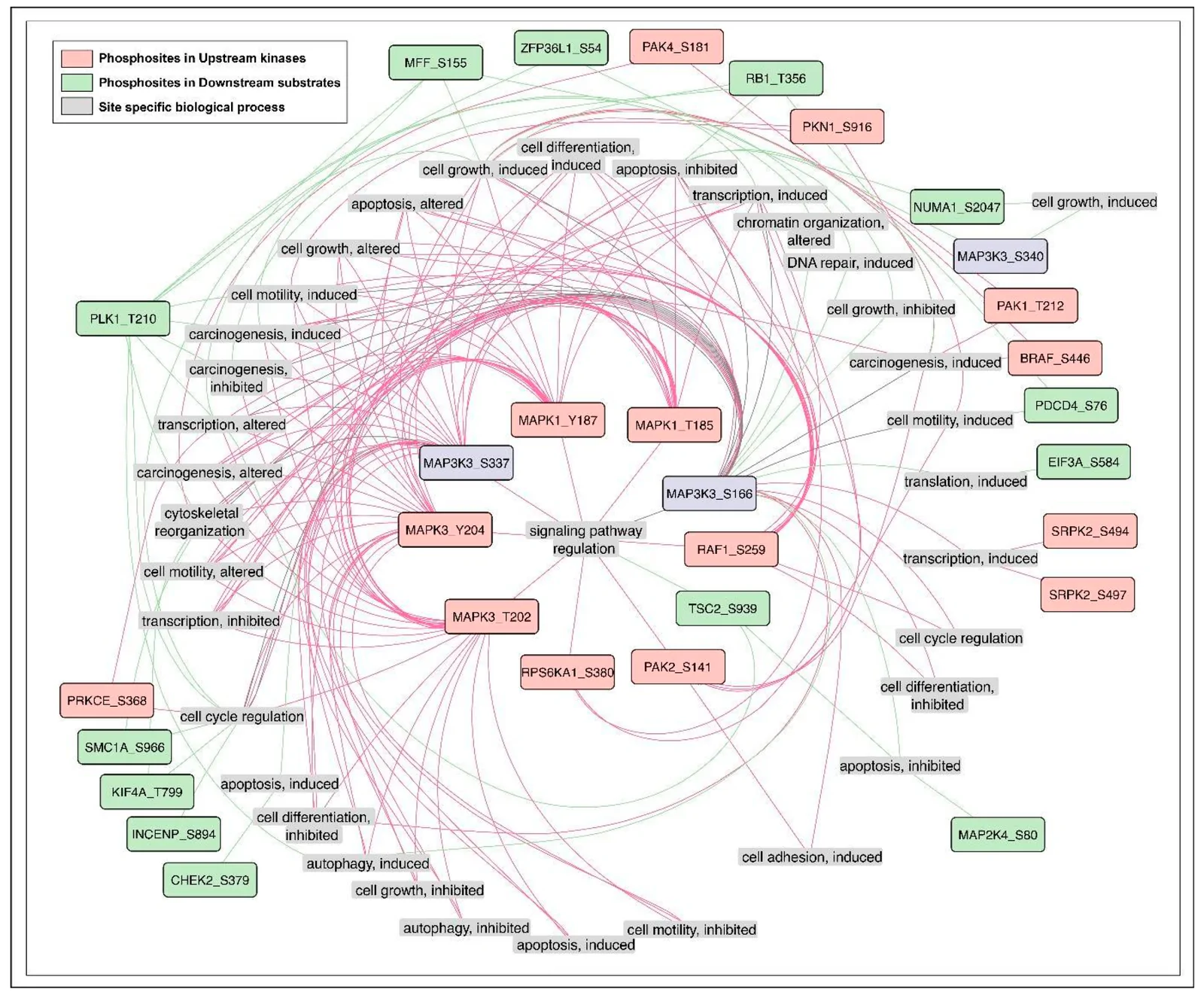
Phosphosignaling network depicting the role of MAP3K3-driven carcinogenesis. Phosphosites in upstream kinase (pink) and phosphosites in downstream substrates (green) and associated biological processes are highlighted. The predominant phosphosites of MAP3K3 are highlighted in purple.

**Figure 8:**
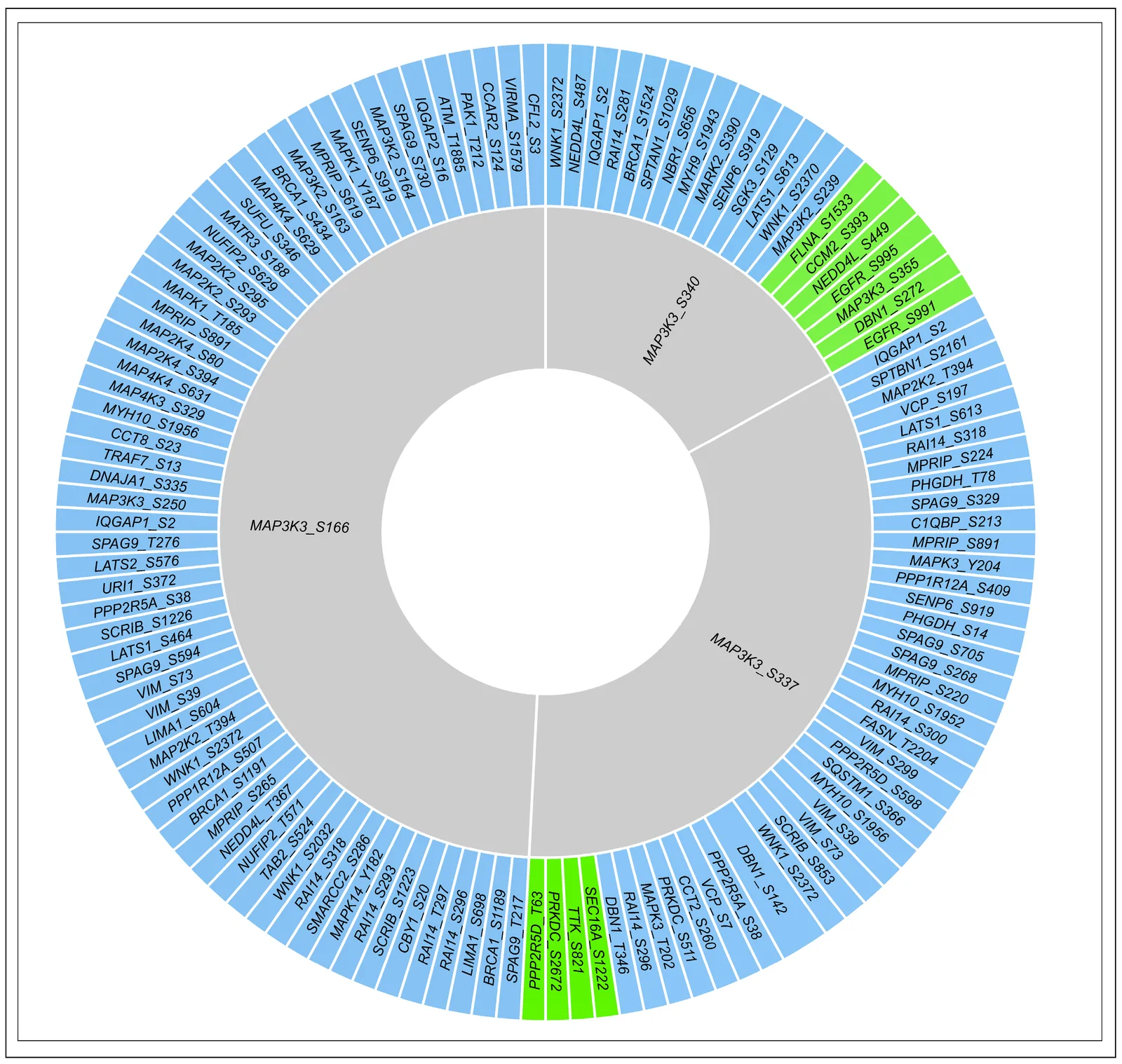
Binary interactors that are positively and negatively co-concordant with the predominant sites of MAP3K3. Binary interactors that are positively co-concordant (blue) and binary interactors that are negatively co-concordant (green). The predominant phosphosites of MAP3K3 are highlighted in grey.

## Data Availability

The data supporting the findings of this study are available in the Supplementary Files.

## Supplementary Materials

The following supporting information can be downloaded at: https://www.mdpi.com/article/10.3390/proteomes14040050/s1.

## Abbreviations

CCM	Cerebral cavernous malformation
FET	Fisher’s exact test
JNKs	c-Jun-N-terminal
MAP3K3	Mitogen-activated protein kinase kinase kinase 3
PoKD	Predominant phosphosites outside the kinase domain
PsOPs	Phosphosites in other proteins
PTM	Post-translational modification
SCCM	Spinal cord cavernous malformation
IIDD	Positive co-concordance
IDDI	Negative co-concordance
